# Supplementary material for: Ensemble-Based Binding Free Energy Profiling and Network Analysis of the KRAS Interactions with DARPin Proteins Targeting Distinct Binding Sites: Revealing Molecular Determinants and Universal Architecture of Regulatory Hotspots and Allosteric Binding
Source: Biomolecules. 2025 Jun 5;15(6):819. doi: 10.3390/biom15060819 (PMC12190860; doi:10.3390/biom15060819)
Supplement: Supplementary file 1 [file biomolecules-15-00819-s001.zip › biomolecules-3679398-supplementary.pdf]

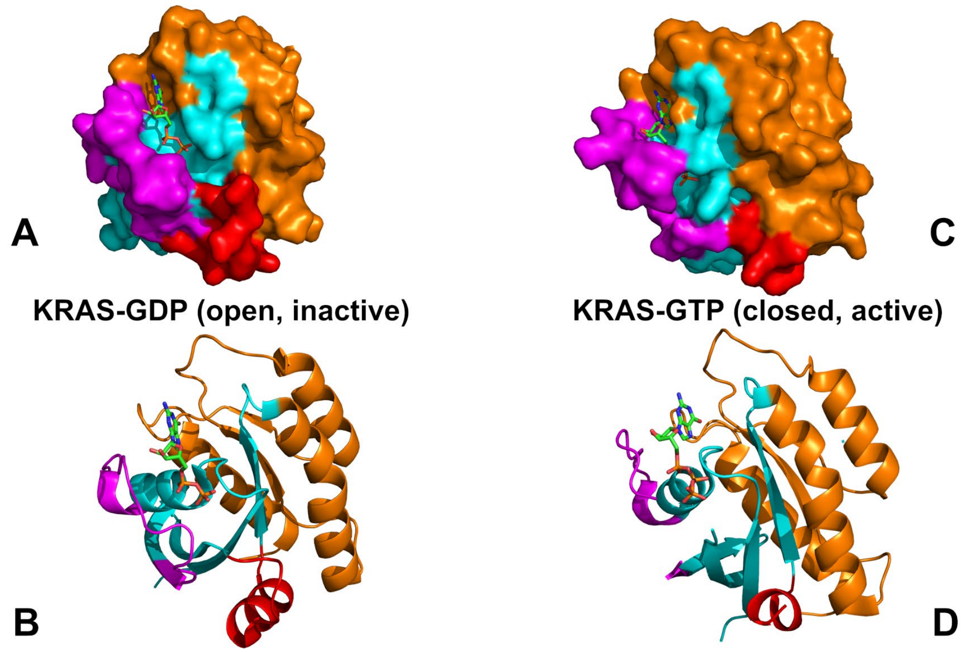

Figure S1. Structural representation of KRAS in its open (state I) and closed (state II) conformations, highlighting the dynamic equilibrium between these states in the GTP-bound form.

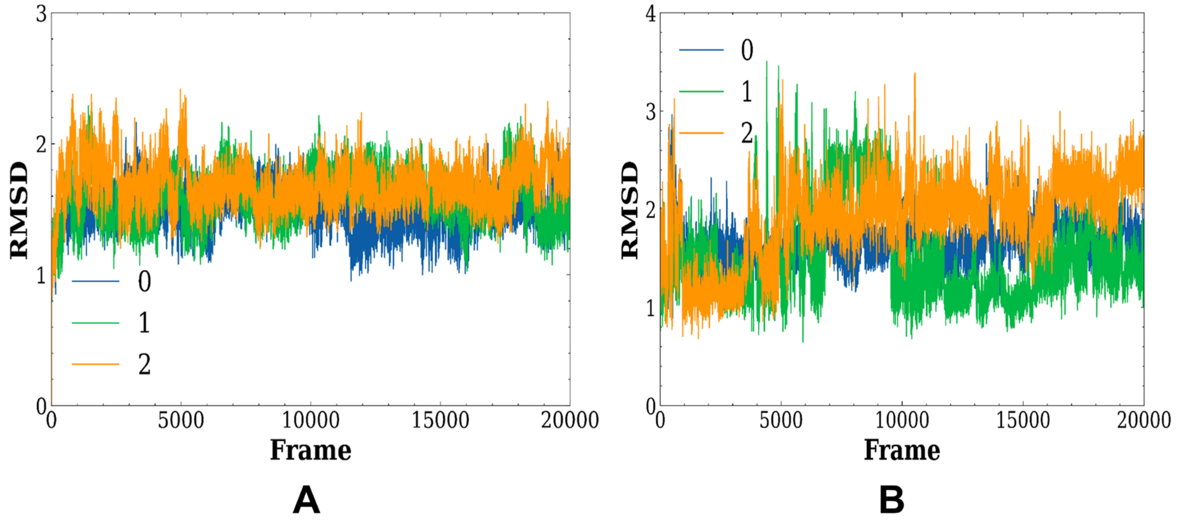

Figure S2. The RMSD profiles for K27-KRAS and K13 -KRAS complexes.

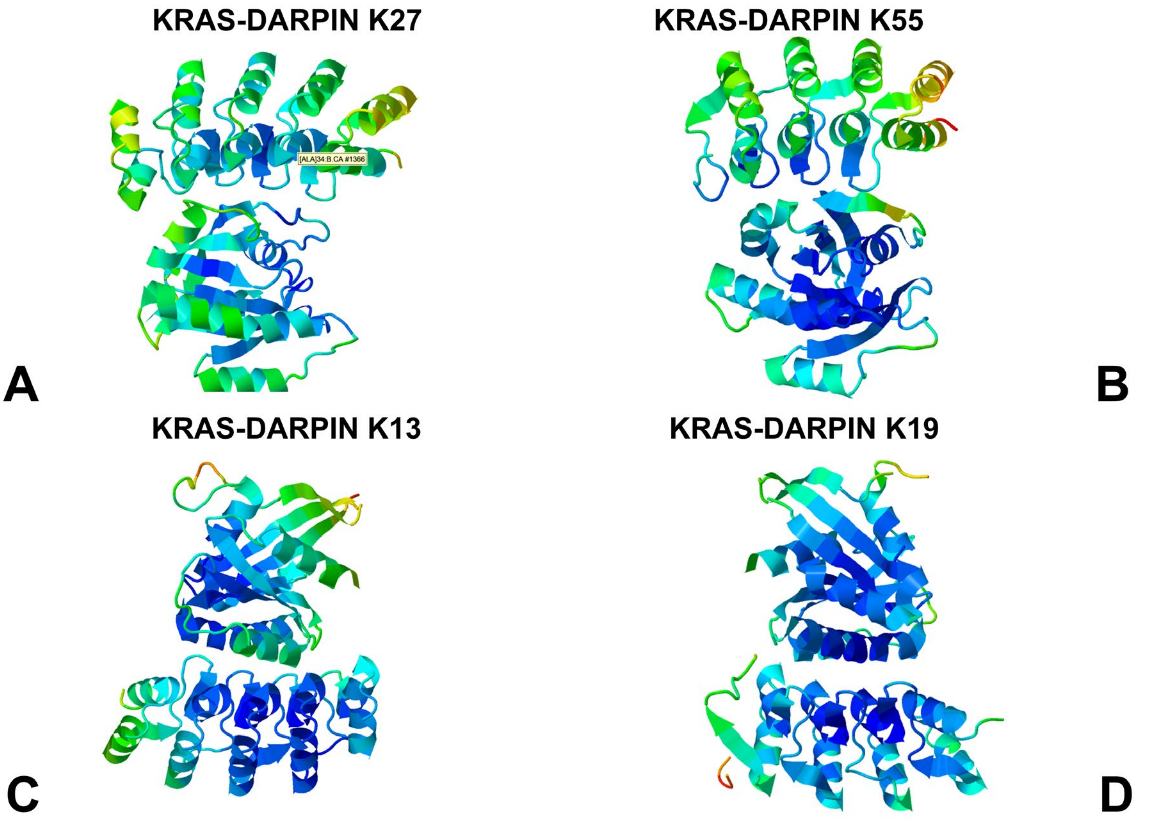

Figure S3. Structural mapping of conformational mobility profiles of KRAS-DARPin 1236 complexes obtained from MD simulations.

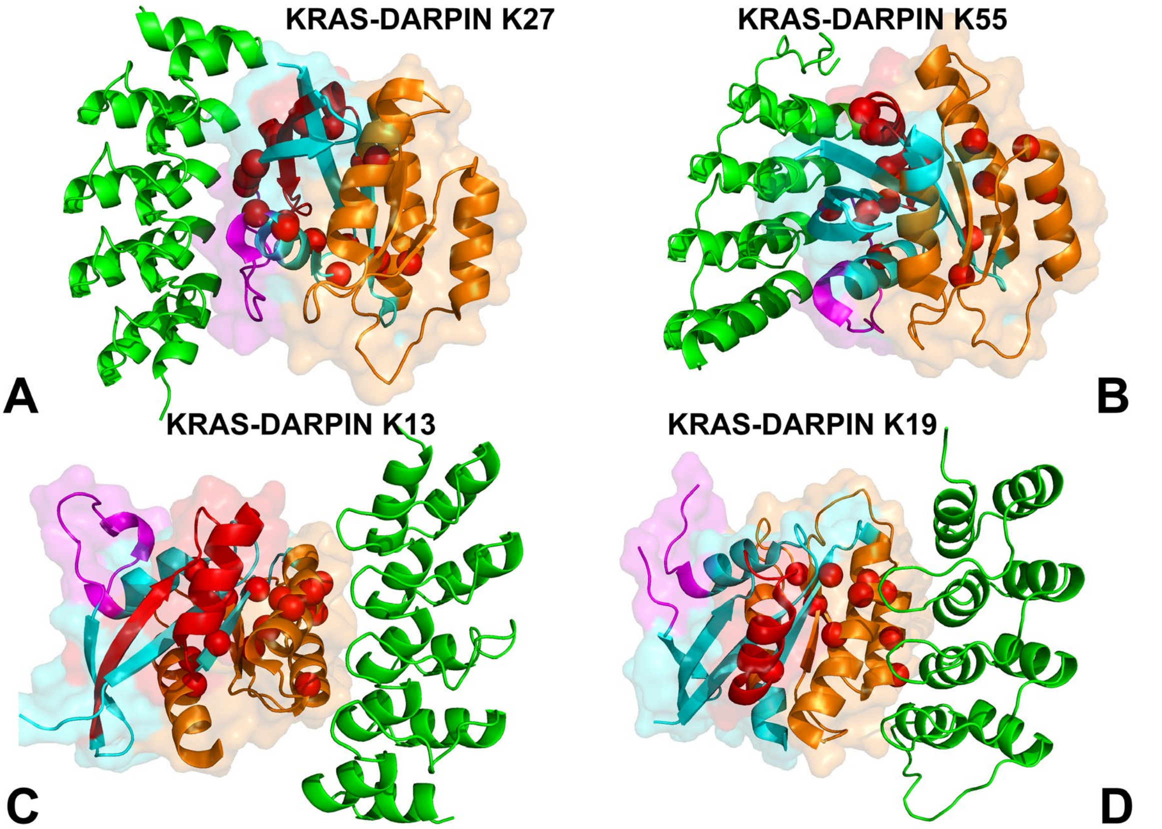

Figure S4. Structural mapping of allosteric com-1237 munication hotspots in KRAS complexes with DARPin proteins.

Table S1. Summary of KRAS-DARPin complexes analyzed in this study.

**KRAS-K27**	**5O2S**	**Targets GDP-bound inactive conformation; stabilizes P-loop and Switch I**	**Acts as a conformational blocker, preventing SOS1-mediated nucleotide exchange**
**KRAS-K55**	5O2T	Mimics natural effectors (e.g., RAF1); binds GTP-bound active state	Stabilizes both Switch I and II, locking KRAS into a signaling-ready geometry
**KRAS-K13**	6H46	Binds to an allosteric lobe (helix α3–loop–α4); minimal involvement of effector interface	Modulates conformational equilibrium without fully immobilizing switch regions
**KRAS-K19**	6H47	Targets the same allosteric lobe as K13 but with greater polar interactions	Introduces new polar constraints, enhancing KRAS specificity and modulating long-range communication
**KRAS-RAF1**	6VJJ	Natural effector binding; engages Switch I and II in the GTP-bound active state	Represents a reference system for understanding effector engagement and KRAS dynamics

Table S2. System setup details for KRAS-DARPIN complexes used in MD simulations.

**PDB**	**SYSTEM**	**PROTEIN HEAVY ATOMS**	**WATER MOLECULES**	**Na$^+$**	**Cl$^-$**	**TOTAL NUMBER OF ATOMS**	**SOLVENT MODEL**	**BUFFR SIZE**	**FORCE FIELD**
5O2S	KRAS G12V-GDP + DARPIN K27	2,338	4,980	10	8	~62,000	TIP3P	12	ff14SB + TIP3P
5O2T	KRAS G12V-GDP + DARPIN K55	2,211	4,812	9	7	~60,000	TIP3P	12	ff14SB + TIP3P
6H46	KRAS G12C-GDP + DARPIN K13	2,396	5,134	11	9	~63,500	TIP3P	12	ff14SB + TIP3P
6H47	KRAS G12C-GDP + DARPIN K19	2,243	5,021	10	8	~62,300	TIP3P	12	ff14SB + TIP3P
